# Physical Activity Promotion in Aging Adults With Rheumatic Diseases: Perspectives From Aging Adults With Lupus

**DOI:** 10.1093/geroni/igaf122.2116

**Published:** 2025-12-31

**Authors:** Sarah Lieber, Sarah Young, Dongmei Sun, Carol Sames, Lisa Mandl, Sara Czaja, M Carrington Reid, Iris Navarro-Millan

**Affiliations:** Hospital for Special Surgery, New York, New York, United States; Weill Cornell Medicine, New York, New York, United States; Hospital for Special Surgery, New York, New York, United States; Upstate Medical University, Syracuse, New York, United States; Hospital for Special Surgery, New York, New York, United States; Weill Cornell Medicine, New York, New York, United States; Weill Cornell Medicine, New York, New York, United States; Weill Cornell Medicine, New York, New York, United States

## Abstract

Aging adults with systemic rheumatic diseases, including systemic lupus erythematosus (SLE), face unique challenges related to physical activity (PA) engagement, including mobility limitations and multicomplexity, and may benefit from tailored approaches to PA promotion. PA may be especially important in aging adults with SLE, who have high rates of cardiovascular disease and multimorbidity, but low rates of PA. In this qualitative study, we elicited perspectives of adults with SLE on PA promotion, including delivered via digital health technologies. Adults ≥50 years of age with SLE took part in semi-structured interviews, drawing in part from a socioecological framework and the technology acceptance model. We obtained self-reported data on sociodemographic and disease characteristics and asked participants to offer insights on PA promotion strategies, particularly related to digital health technology. We performed a thematic data analysis. Among 30 participants with SLE, who were 93.3% female with a mean age of 62.3 years, themes included PA intervention suggestions, digital literacy and access, and usefulness and ease of use of technology in the context of PA promotion. Participants recommended incorporating friends and family into PA promotion efforts. We identified interest among participants in tailored PA strategies for SLE, potentially incorporating digital health technology and social connection. Designing, testing, and disseminating tailored interventions to enhance lifestyle changes, including regular PA, among aging adults with SLE, as well as other systemic rheumatic diseases, could lead to improved health and wellbeing in conjunction with disease-directed therapies in this growing patient population.

